# Genotypic Differences in the Effect of P Fertilization on Phytic Acid Content in Rice Grain

**DOI:** 10.3390/plants9020146

**Published:** 2020-01-23

**Authors:** Ayaka Fukushima, Ishara Perera, Koki Hosoya, Tatsuki Akabane, Naoki Hirotsu

**Affiliations:** 1 Graduate School of Life Sciences, Toyo University, 1-1-1 Izumino, Itakura-machi, Oura-gun, Gunma 374-0193, Japan; s39101900193@toyo.jp; 2 Grain Legumes and Oil Crops Research and Development Centre, Department of Agriculture, Angunakolapelessa 82220, Sri Lanka; isharauip@gmail.com; 3 Faculty of Life Sciences, Toyo University, 1-1-1 Izumino, Itakura-machi, Oura-gun, Gunma 374-0193, Japan; s19101601054@toyo.jp (K.H.); s19101600929@toyo.jp (T.A.)

**Keywords:** inorganic P, P fertilizer, phytic acid, rice

## Abstract

Phytic acid (PA) prevents the absorption of minerals in the human intestine, and it is regarded as an antinutrient. Low PA rice is beneficial because of its higher Zn bioavailability and it is suggested that the gene expression level of *myo*-inositol 3-phosphate synthase 1 (*INO1*) in developing grain is a key factor to explain the genotypic difference in PA accumulation among natural variants of rice. P fertilization is also considered to affect the PA content, but it is not clear how it affects *INO1* gene expression and the PA content in different genotypes. Here, we investigated the effect of P fertilization on the PA content in two contrasting rice genotypes, with low and high PA accumulation, respectively. Based on the results of the analysis of the PA content, inorganic P content, *INO1* gene expression, and xylem sap inorganic P content, we concluded that the effect of P fertilization on PA accumulation in grain differed with the genotype, and it was regulated by multiple mechanisms.

## 1. Introduction

Many people living in developing countries have a higher risk of malnutrition due to Zn deficiency, as they mainly take micronutrients from cereals, such as legumes, wheat, and rice. Atmospheric carbon dioxide (CO_2_) concentration is increasing and expected to reach 700 ppm by the end of this century [1]. Elevated CO_2_ (e[CO_2_]) causes a reduction in the mineral content in staple crops [2]. In the future, Zn deficiency is expected to expand globally, especially in developing countries, due to e[CO_2_]. Phytic acid (*myo*-insitol-1,2,3,4,5,6-hexakisphate; PA) is the storage form of P in cereal grains; it accounts for 75% of total P in grains [3]. Phytic acid is also known to chelate with minerals, such as Fe, Mg, Ca, K, and Zn. It prevents the absorption of minerals in the human intestine and is regarded as an antinutrient. Research conducted using a suckling rat pup model showed a negative correlation between dietary PA and Zn absorption from the grains of maize, rice, and barley [4]. In addition, PA inhibits enzymes needed for protein degradation and disturbs proteolysis in the stomach and small intestine [5]. Ruminants, such as cows, secrete phytase, an enzyme digesting PA, but humans lack phytase and therefore cannot digest PA. Thus, reducing the PA content in cereals is essential to overcome Zn deficiency.

The regulation of PA content has to take into account the P status in plants, P absorption from the roots, and remobilization from plant organs. P transporters are known to be involved in the uptake of inorganic P (Pi) from the root and transport to plant organs. So far, 13 Pi transporters belonging to the PHT1 family have been identified in rice [6,7]. OsPT8 known as a high affinity Pi transporter is essential for Pi translocation from vegetative organs into rice gain [8]. SULTR-like phosphorus distribution transporter (SPDT) controls the allocation of P to the grains [9]. These findings suggest that it might be possible to control the PA content by manipulating P transporter.

To identify the biosynthetic pathway of PA and reduce the PA content in the grain, low phytic acid (*lpa*) mutants of wheat [10], maize [11,12,13], soybean [14,15], barley [16,17,18], and rice [19] have been used. These mutants have disrupted PA biosynthesis genes and exhibit low PA accumulation in the grains; however, in most cases, these mutants showed a significant reduction in germination and yield [20]. On the contrary, mutants that repress *INO1* with an 18-kDa oleosin promoter showed an approximately 70% reduction in the PA content, with no negative effects on plant growth [21]. Similarly, no undesirable yield reduction was observed in *lpa* mutants of barley [22] and soybean [23]. Developing stable *lpa* mutants without yield loss is indispensable to overcome malnutrition.

P is one of the most essential elements for plant growth. P fertilizer is indispensable in practical agriculture and therefore P application cannot be reduced [24]. The applied P is absorbed from the root and remobilized to the shoot, and then PA is synthesized in developing seeds using the transported P [25]. It has been reported that the PA content is affected by the amount of supplied P in various crops [26,27,28], including rice [29]. In a low-phytate soybean line, which derived from a cross of the normal-phytate Japanese cv. Tanbakuro and the low-phytate line CX1834, no negative effects were reported on plant growth and yield, leaf photosynthesis, and nitrogen fixation by different levels of P fertilizer application [30]. These results suggest that the *lpa* mutant can exhibit a low PA phenotype without any loss of growth-related performances at various levels of P fertilizer application. Thus, information on the effect of P fertilizer on seed PA content and yield in the *lpa* mutant is accumulating; however, it is not clear how P fertilizer affects the PA content in natural genotypic variants.

Previously, we examined variations in the PA content in 69 accessions of the World Rice Core Collection (WRC) and identified WRC 5 and WRC 6 as cultivars with the lowest and highest PA content in the collection [31]. We then compared WRC 5 and WRC 6 to identify the molecular determinant of the natural variation in the PA content in rice. The results suggested the gene expression level of *myo*-inositol 3-phosphate synthase 1 (*INO1*) was the genetic basis explaining the natural variation in PA accumulation in rice [32]. Interestingly, DNA sequences of the coding and promoter region (1000 bp) of the *INO1* gene were identical between WRC 5 and WRC 6. This suggests there are different regulation mechanisms of PA content besides DNA mutation of the biosynthesis gene. To elucidate these regulation mechanisms, WRC 5 and WRC 6 will be useful cultivars. In this study, we evaluated the effect of P fertilization on the PA content in WRC 5 and WRC 6 to clarify how the PA content is regulated under a different P status in natural variants of rice.

## 2. Results

### 2.1. Differences in Yield-Related Traits

#### 2.1.1. Effect of P Fertilization in Low and High PA Rice

Yield-related traits of WRC 5 and WRC 6 are shown in Table 1. There were no significant differences in the panicle number and panicle weight between low and high PA plants. The panicle length of WRC 6 was significantly shorter than that of WRC 5. The panicle weight of WRC 5 was slightly higher than that of WRC 6. No significant difference was observed in the total yield per plant.

#### 2.1.2. Effect of the Amount of P Fertilizer on Initial Growth and Yield

To investigate the effect of P fertilizer on yield-related traits, the panicle weight and number (Table 2) and initial growth at the seedling stage (Table 3) were measured in WRC 5 and WRC 6. No significant differences were observed in WRC 5 and WRC 6.

### 2.2. Effects of the Time of P Fertilizer Application 

**Figure 1:**
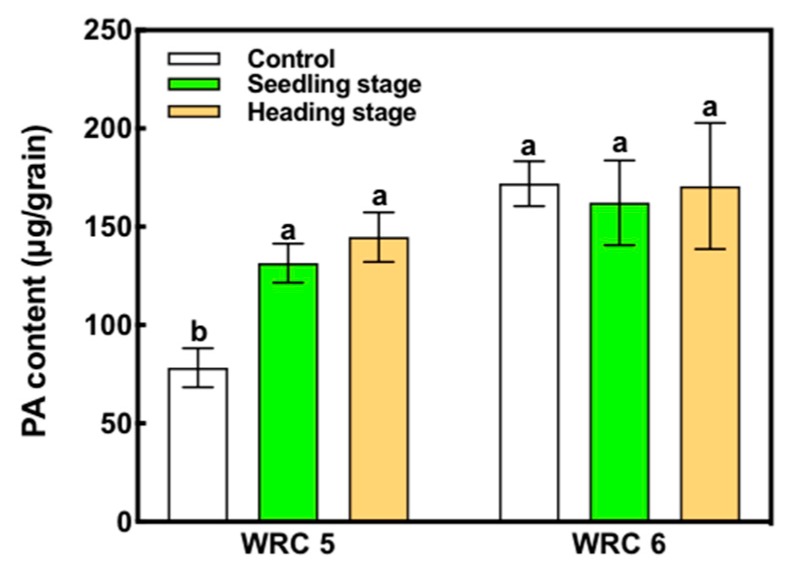
Effect of the time of P fertilizer application on the PA content in WRC 5 and WRC 6. White, green, and orange indicate control, P applied in the seedling stage, and P applied in the heading stage, respectively. Each value represents the mean ± SD of four replicates. Different letters indicate statistical differences (Tukey’s HSD test, *p* < 0.05).

WRC 5 showed a lower PA content than WRC 6 under the control condition (Figure 1). When P fertilizer was applied at the seedling stage, WRC 5 presented increased PA content compared with that of the control, whereas WRC 6 presented no increase. When P fertilizer was applied in the heading stage, there was no significant difference in the PA content compared with that when P fertilizer was applied in the seedling stage.

### 2.3. Variation in the PA Content with Change in the P Concentration in the Soil

**Figure 2:**
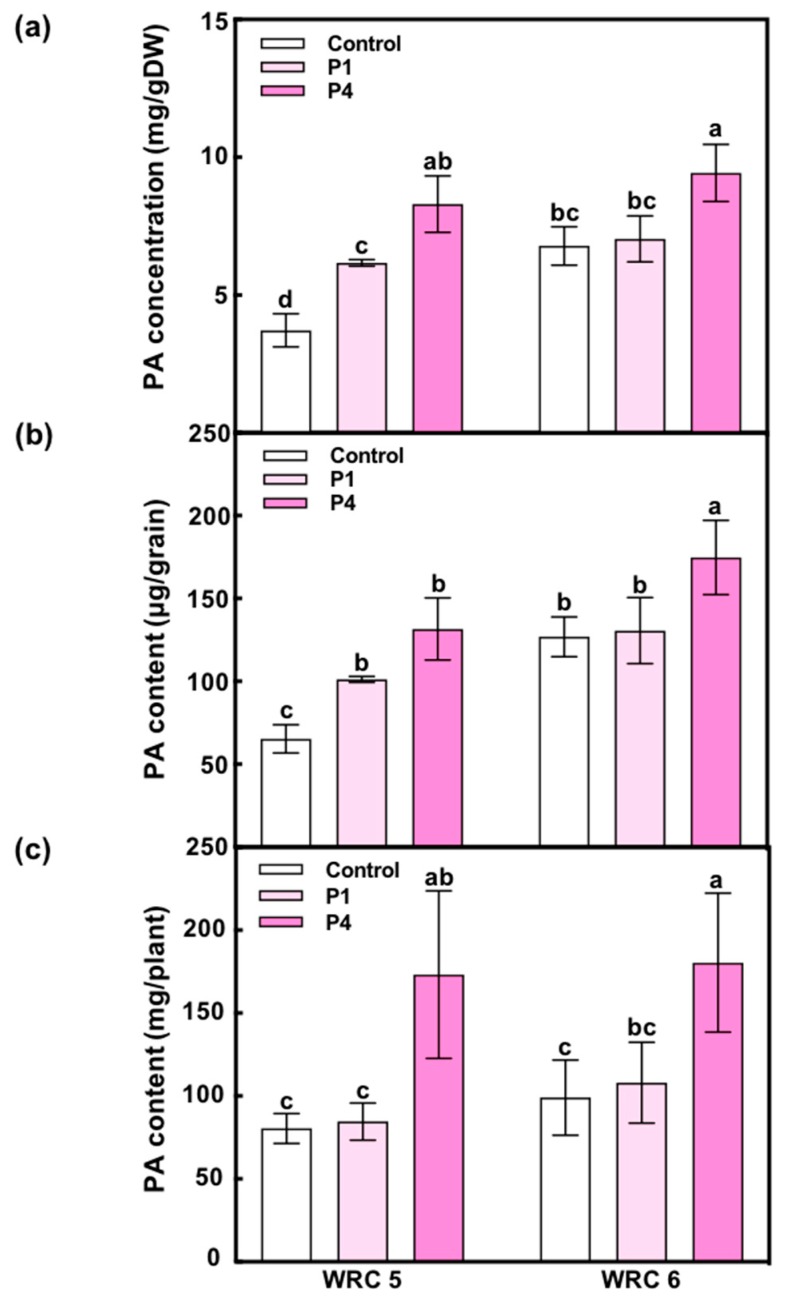
Effect of the amount of P fertilizer on PA accumulation in WRC 5 and WRC 6. (**a**) PA concentration per gram dry weight (mg/gDW), (**b**) PA content per grain (μg/grain), (**c**) PA content per plant (mg/plant). White, pale pink, and pink indicate control, P1 treatment, and P4 treatment, respectively. Each value represents the mean ± SD of four replicates. Different letters indicate statistical differences (Tukey’s HSD test, *p* < 0.05).

To examine the effect of the amount of P fertilizer on PA accumulation in the grain, we applied different amounts of P fertilizer at the seedling stage and measured the PA concentration and content in harvested grains (Figure 2). In the control, WRC 6 showed a higher PA concentration and content than WRC 5 as expected (Figure 2a,b). We observed that the PA concentration and content in both cultivars increased under P4 treatment.

### 2.4. PA-P and Pi Content

**Figure 3:**
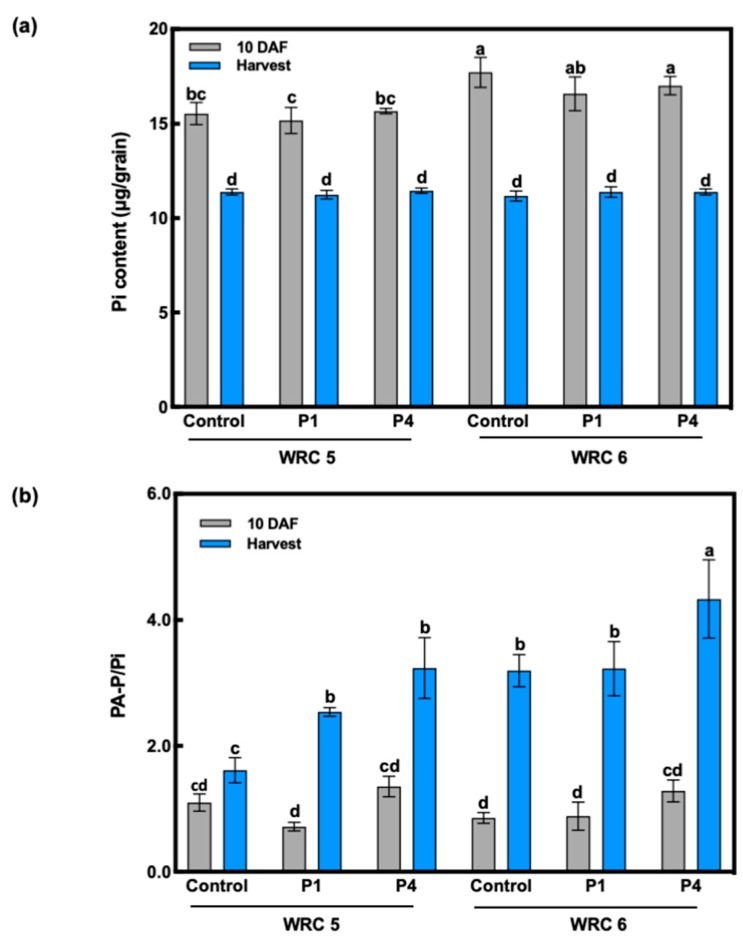
Effect of the amount of P fertilizer on Pi and PA-P/Pi in WRC 5 and WRC 6. **(a**) Pi content per gram dry weight (μg/grain) and (**b**) PA-P/Pi ratio at 10 DAF and harvest. Gray and blue represent the value at 10 DAF and harvest, respectively. Each value represents the mean ± SD of four replicates. Different letters indicate statistical differences (Tukey’s HSD test, *p* < 0.05).

The Pi content and PA-P/Pi ratio at 10 days after flowering (DAF) and at harvest are shown in Figure 3. We found that the Pi content showed no response to P fertilizer treatment (Figure 3a). WRC 6 showed a significantly higher Pi content at 10 DAF than WRC 5. The PA-P/Pi ratio presented no significant difference between P treatments or genotypes at 10 DAF (Figure 3b). The PA-P/Pi ratio at harvest was increased under P4 treatment and was associated with the PA content per grain (Figure 2b and Figure 3b).

### 2.5. INO1 Expression and PA Content at 10 DAF

**Figure 4:**
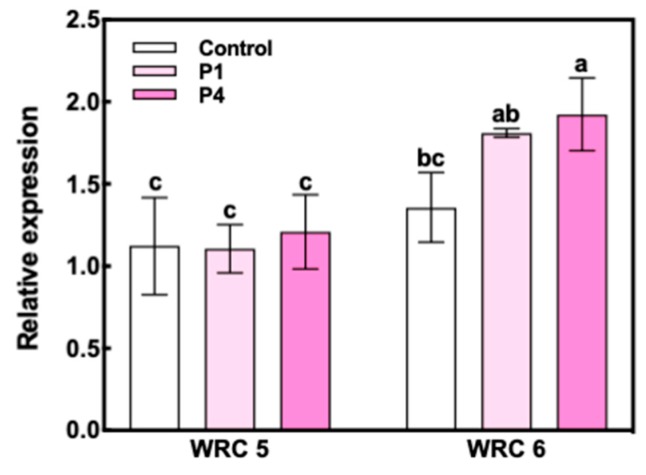
Relative gene expression of *INO1* at 10 DAF in WRC 5 and WRC 6. White and pink indicate the control and P treatment, respectively. Each value represents the mean ± SD of three replicates. Different letters indicate statistical differences (Tukey’s HSD test, *p* < 0.05).

**Figure 5:**
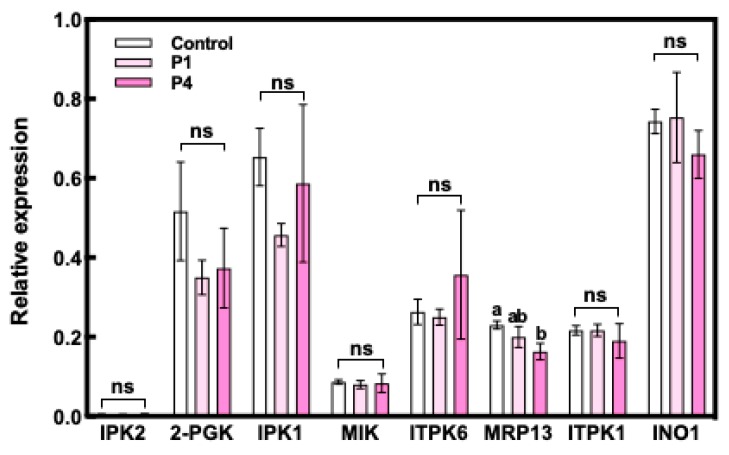
Expression level of PA biosynthesis genes in Nipponbare. White, pale pink, and pink indicate the control, P1 treatment, and P4 treatment, respectively. Each value represents the mean ± SD of three replicates. Different letters indicate statistical differences (Tukey’s HSD test, *p* < 0.05).

The expression of *INO1* at 10 DAF was significantly increased by P fertilizer treatment in WRC 6, whereas WRC 5 showed no response to P fertilizer treatment (Figure 4). We also investigated the expression levels of eight PA biosynthesis genes in Nipponbare (Figure 5). Among these eight genes, no significant differences in the expression level were observed except for the multidrug resistance-associated protein 13 (*MRP13*) gene, which showed a significant decrease under the P4 treatment.

### 2.6. Short-Term Response of Pi Uptake in WRC 5 and WRC 6

**Figure 6:**
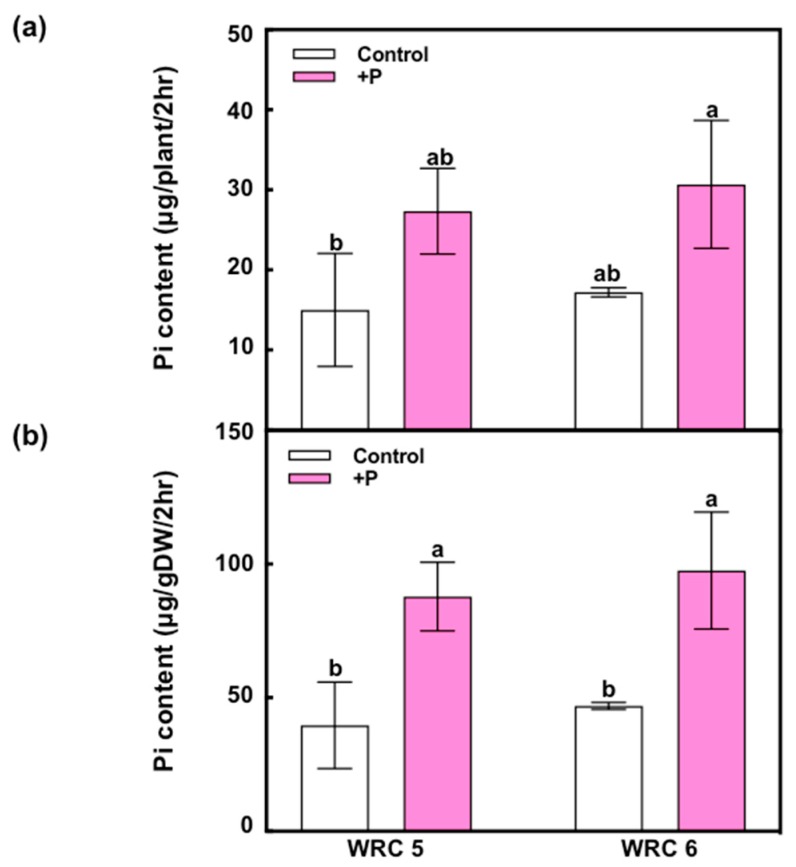
Short-term response of Pi uptake using the xylem sap analysis in WRC 5 and WRC 6. (**a**) Pi content in xylem sap oozed out from one seedling for 2 h (μg/plant/2 h) and (**b**) Pi content per root DW (μg/gDW/2 h). White and pink indicate the control and P treatment, respectively. Each value represents the mean ± SD of three replicates. Different letters indicate statistical differences (Tukey’s HSD test, *p* < 0.05).

To evaluate Pi uptake on WRC 5 and WRC 6 in the initial growth stage, we measured the Pi content in xylem sap using the molybdenum blue method (Figure 6). When additional P fertilizer was applied, the Pi content in xylem sap per root dry weight (μg/gDW/2 h) was significantly increased, whereas there was no significant difference between WRC 5 and WRC 6.

## 3. Discussion

### 3.1. Effect of P Fertilizer on the PA Content

To develop *lpa* mutants, DNA mutations in the PA biosynthesis genes have been used. These *lpa* mutants helped reveal the mechanism of PA biosynthesis; however, *lpa* mutant rice often exhibited low plant biomass and yield compared with those of wild type [20]. Therefore, the use of *lpa* rice mutants in commercial agriculture is not very competitive. In our study, two contrasting rice cultivars selected in terms of PA content from natural variation in the rice germplasm did not show any significant difference in the initial growth (Table 3) and yield-related traits (Table 1). P fertilization did not affect the panicle weight and yield in both WRC 5 and WRC 6 (Table 2). It suggested that the sensitivity of P application in terms of plant growth and yield was similar between WRC 5 and WRC 6. Thus, these contrasting lines are effective in analyzing the mechanism of P fertilizer response of PA synthesis without consideration of the plant yield and growth differences.

In rice, P is accumulated in the leaves until the heading stage, and then transported to the stem in the heading stage. Finally, P is stored in the grain at maturity [33]. According this P translocation system, we expect that rice would show a different response of PA content with the time of P fertilizer application. Our study showed that the PA content increased with P fertilizer in WRC 5, irrespective of the time of P fertilizer application (Figure 1). Moreover, the PA content did not change in WRC 6 with P fertilizer. These results indicate that the effect of P fertilizer on the assimilation and translocation of P is not stage dependent but differs with the genotype.

When a higher amount of P was applied, the PA content increased in both cultivars (Figure 2a). Because the grain yield was not affected by higher P (P4) application, the PA content per grain (Figure 2b) or per plant (Figure 2c) also increased, indicating that PA biosynthesis was upregulated by P application. On the contrary, the PA content under the control treatment differed with the genotype; WRC 5 showed a significantly lower PA content than WRC 6. This result suggests that WRC 5 and WRC 6 have a different responsiveness toward soil P, and WRC 6 might be sensitive to PA accumulation under lower soil P conditions. The Pi content in WRC 6 at 10 DAF was higher than that in WRC 5 (Figure 3a), and this might be due to the higher PA content in WRC 6. Interestingly, the Pi content at 10 DAF did not respond to P fertilizer application, and Pi influx in the early development stage of grain might determine the genotypic differences in the PA content.

### 3.2. Effect of P Fertilizer on PA Biosynthesis

While the PA content varied in response to P application, the Pi content remained constant and was not affected by the P application and genotype (Figure 3a). The Pi content in the grain was found to be regulated at a constant level. While the Pi content was constant, the ratio of PA-P to Pi responded to P fertilizer, and it was associated with the PA content (Figure 3b). This suggests that the biosynthesized PA using influxed Pi determines the PA response to P application. To assess the PA biosynthesis level, we analyzed *INO1* gene expression, which we previously reported as the key determinant that explains genotypic differences between WRC 5 and WRC 6 [32]. The expression of *INO1* differed with genotype; WRC 6 responded to P fertilizer, whereas WRC 5 did not show any response to P fertilizer (Figure 4). This differential response to P fertilizer might be one of the key factors explaining the genotypic difference in the PA content and the difference in P responsiveness. Simultaneously, while *INO1* showed no response to P fertilizer, the PA content was stimulated by P fertilizer in WRC 5 (Figure 2b), suggesting there might be other factor(s), besides *INO1* gene expression, that regulate the PA biosynthesis response to P fertilizer.

### 3.3. Other Factors Regulating Grain PA Content

The biosynthesis of PA continues until 25 DAF [32,34]; we investigated the expression level of other genes related to PA synthesis identified from the Nipponbare genome database [35]. Seven genes, namely, inositol 1, 3, 4-trisphosphate 5/6-kinase 2 (*ITPK2*), 2-phospho-glycerate kinase (*2-PGK*), inositol-pentakisphosphate 2-kinase 1 (*IPK1*), myo-inositol kinase (*MIK*), inositol 1, 3, 4-trisphoshate 5/6-kinase *2* (*ITPK6*), inositol 1, 3, 4-triskisphosphate 5/6-kinase 1 (*ITPK1*), and *INO1* showed no response to P fertilizer treatment in Nipponbare (Figure 5). We could not compare these gene expression levels in WRC 5 and WRC 6 directly because WRC 5 and WRC 6 might have different DNA sequences in the priming site of the polymerase chain reaction (PCR) and this interferes with the accurate comparison of the gene expression level. Although we should further analyze gene expression using WRC 5 and WRC 6, the results indicated that the PA content response to P fertilizer might not be regulated by PA biosynthesis in Nipponbare.

There are three potential steps that determine the grain PA content, namely, uptake of soil-Pi, translocation of Pi within the plant body, and biosynthesis and accumulation of PA in the grain. To analyze Pi uptake by the root system and Pi translocation capacity from the root to shoot, we analyzed the xylem sap in seedlings grown by hydroponic culture (Figure 6). The Pi content in xylem sap increased with P fertilizer application, but there was no difference between WRC 5 and WRC 6. These results indicate that the Pi uptake and translocation in the seedling stage are stimulated by P fertilizer; this additional Pi might be used for additional PA biosynthesis. Contrarily, we could not find any genotypic difference in the Pi content in xylem sap in the seedling stage. Further investigation of xylem sap collected at various growth stages will be required to asses Pi uptake and Pi translocation capacities. Recently, SPDT is reported to regulate Pi distribution between the leaves and grains [9]. A comprehensive analysis throughout Pi uptake, translocation, and grain accumulation will be important to further elucidate the effect of P fertilizer on PA accumulation in grains.

## 4. Materials and Methods

### 4.1. Plant Materials

We obtained WRC accessions from the National Agriculture and Food Research Organization (NARO) Genebank in Tsukuba, Ibaraki, Japan. The selected WRC 5 (NABA) and WRC 6 (PULUIK ARANG) lines were grown in an outside paddy field or in 1/5000 Wagner pots filled with soil (Bon-sol #2; Sumitomo Chemical, Tokyo, Japan; containing 1.5 g each N, P, and K) in Itakura, Gunma, Japan (36°13′23″N 139°36′37″E) in 2018 to 2019. First, to compare the effect of the time of application of P fertilizer, 1 g of P fertilizer (as Ca(H_2_PO_4_)_2_) containing 0.26 g of P was applied into the pots at the seedling stage or heading stage in 2018. Second, we applied different amounts of P fertilizer, 1 g (P1) and 4 g (P4), containing respectively 0.26 and 1.1 g P at the seedling stage in 2019. Developing (10 DAF) and matured (30 DAF) grains were used for further analysis. In the pot experiments, 2 plants were planted in a pot and we used 2 pots to generate 4 replicates. Rice plants grown in the outside paddy field were used for the analysis of yield-related traits. Panicle number, 1000 seed weight, and total yield per plant were analyzed in 5 replicates and panicle length and weight were analyzed in 10 replicates.

### 4.2. Determination of the PA and Pi Content

The Pi and PA content were determined using the enzymatic method [36]. Pi and PA were extracted for 17 h in 0.66 M HCl from one grain of brown rice, and then the Pi and PA content was determined colorimetrically using the Phytic Acid (Phytate)/Total Phosphorus kit (K-PHYT; Megazyme International, Wicklow, Ireland) according to the manufacturer’s instruction after neutralization. This kit measures Pi released from the extracted grain sample after treatment with phytase and alkaline phosphatase. The free Pi content was estimated from samples not treated with phytase.

### 4.3. Analysis of Gene Expression

Developing ovaries collected at 10 DAF were used for gene expression analysis. RNA was extracted using the cetyl trimethyl ammonium bromide method from approximately 6–7 frozen ovaries. First strand cDNA was synthesized from 5 ng of total RNA using the Primer Script RT Reagent kit (PR037A; Takara Bio Inc., Shiga, Japan) according to the manufacturer’s instructions. Quantitative RT-PCR was performed with the LightCycler system (Light Cycler 480, Roche Diagnostics, Basel, Switzerland) using Universal Probe Library (UPL, Roche Diagnostics, Basel, Switzerland). *GAPDH* was used as the reference gene, and the relative expression levels were calculated using the 2^−ΔΔCT^ method [37]. The primers and probes are listed in Table 4.

### 4.4. Analysis of xylem sap

Rice was grown for 4 weeks hydroponically in a growth chamber as described by Nagai et al. [38]. The shoots were cut 3–4 cm above the shoot meristem; the section was covered with silicon tube containing glass wool, and then xylem sap was collected for 2 h (10:00–12:00 h). The glass wool was transferred into an ultrafiltration filter tube (Nanosep, ODGHPC34; Pall Corporation, NY, USA) and centrifuged at 17 800 g for 3 min. Xylem saps were stored at –80 °C. Xylem sap diluted 10 times with distilled water was used to determine the Pi content. Diluted saps (50 μL) were treated with 25 μL of color reagent containing 10% (w/v) ascorbic acid, 97% (w/w) H_2_SO_4_, and 5% (w/v) ammonium molybdate. After incubation at 40 °C for 1 h, the absorbance of the sample at 655 nm was measured using a microplate spectrophotometer (xMark, Bio-Rad, Hercules, CA, USA). A P calibration curve was prepared using P standard solution.

### 4.5. Data Analysis

We conducted statistical analyses using JMP (SAS Institute, Cary, NC, USA) software. Tukey’s HSD test and student’s *t*-test were used to determine significant differences between means at *p* < 0.05.

## 5. Conclusions

In this study, we investigated how P fertilization affects the PA content in rice. WRC 5 showed a lower PA content than WRC 6, and the Pi content at 10 DAF might be related to this genotypic difference in the PA content. In both genotypes, the PA content increased with P fertilizer treatment; this might be due to the stimulation of Pi uptake and translocation. On the contrary, the *INO1* gene expression responded to P fertilizer only in WRC 6, and the response of PA biosynthesis differed with the genotype. It can be concluded that the PA content is regulated by multiple mechanisms, and the response of plants to P fertilizer differed with the genotype. Further comprehensive investigation is required to understand the regulation mechanisms of PA accumulation by different P fertilizer treatments.

## Figures and Tables

**Table 1:** Yield-related traits in WRC 5 and WRC 6. Data are shown as the mean ± SD of 5–10 replicates. Data with asterisks indicate a significant difference between WRC 5 and WRC 6 (Student’s *t*-test, ***p < 0.001).

WRC No.	Panicle Number	Panicle Length (cm)	Panicle Weight (g)	1000 Seed Weight (g)	Total Yield per Plant (g)
WRC 5	16.6 ± 1.1	24.7 ± 2.0	2.8 ± 0.5	20.2 ± 0.7	35.3 ± 4.3
WRC 6	15.2 ± 1.1	20.3 ± 0.7	2.5 ± 0.3	23.4 ± 0.6	36.8 ± 2.2
	ns	***	ns	***	ns

**Table 2:** Panicle number and total yield per plant of WRC 5 and WRC 6. Data are shown as the mean ± SD of four replicates. Different letters indicate statistical differences (Tukey’s HSD test, *p* < 0.05).

Cultivar	Treatment	Panicle Number	Total Yield per Plant (g)
WRC 5	Control	8.8 ± 0.5 ^ab^	19.9 ± 3.6 ^ab^
	P1	6.3 ± 1.0 ^b^	13.3 ± 1.7 ^b^
	P4	10.0 ± 1.4 ^a^	20.5 ± 4.5 ^a^
WRC 6	Control	9.0 ± 2.7 ^ab^	14.6 ± 2.6 ^ab^
	P1	9.0 ± 0.8 ^ab^	16.1 ± 2.5 ^ab^
	P4	10.0 ± 1.4 ^a^	17.9 ± 3.4 ^ab^

**Table 3:** Initial growth of WRC 5 and WRC 6 at the seedling stage. Data are shown as the mean ± SD of four to five replicates. ns indicates that there was no significant difference between WRC 5 and WRC 6 (Student’s *t*-test).

	Shoot		Root	
	Weight (g)	Length (mm)	Weight (g)	Length (mm)
WRC 5	0.76 ± 0.087	333 ± 39	0.52 ± 0.15	151 ± 23
WRC 6	0.73 ± 0.033	306 ± 24	0.62 ± 0.037	171 ± 17
	ns	ns	ns	ns

**Table 4:** List of primer and probes used in the gene expression analysis.

Gene	Forward (5′–3′)	Reverse (5′–3′)	Probe#
*INO1*	CAGGGTCGGGAGCTACAA	AAGGTCATCAGGGTTCACCA	#52
*ITPK1*	AAGGTGAAGAGCTTCCTCCAG	TTCAGAGAGAGGACGAGTCTCA	#72
*MRP13*	GCACTAGCAAGCAAGACCGTA	TGATATGACCATCCTTAAGAACCA	#146
*ITPK6*	GGCAAACCTCTTACATTCAACAGT	TTGCACCTCGTTTTGCAG	#68
*MIK*	CGATGAAGGAGTTTGTGTCACT	GAAGATCCTTGGCACTTAGCC	#120
*IPK1*	GCTCTTCTAATTTCTGACCACACA	GCCTTTATCTCCACTGCTATGC	#38
*2-PGK*	TGAAGAAAGAGATATGCACGAGA	CAGATCGTCGTATTCCTCGTC	#22
*ITPK2*	GTACGCCCTCACCAAGAAGA	CAATTGCTACAAGATTAATTCCCTTC	#41
*GAPDH*	GCTGCTGCTCACTTGAAGG	AAACATCGGAGCATCTTTGC	#142
